# CAR-T cell signaling dynamics, rational design principles and artificial intelligence for next-generation chimeric antigen receptors

**DOI:** 10.3389/fimmu.2026.1893076

**Published:** 2026-07-08

**Authors:** Xin Liu, Fangjia Tong, Jiayi Zhang, William Gradishar, Huiping Liu, Rongfu Wang

**Affiliations:** 1 Department of Medicine, Keck School of Medicine, University of Southern California, Los Angeles, CA, United States; 2 Department of Pharmacology, Northwestern University Feinberg School of Medicine, Chicago, IL, United States; 3 Division of Hematology and Oncology, Department of Medicine, Northwestern University Feinberg School of Medicine, Chicago, IL, United States; 4 Robert H. Lurie Comprehensive Cancer Center, Northwestern University Feinberg School of Medicine, Chicago, IL, United States; 5 Biohub, Chicago, IL, United States; 6 Norris Comprehensive Cancer Center, Keck School of Medicine, University of Southern California, Los Angeles, CA, United States; 7 Department of Pediatrics, Children’s Hospital Los Angeles, Keck School of Medicine, University of Southern California, Los Angeles, CA, United States

**Keywords:** artificial intelligence (AI), cancer immunotherapy, CAR-T, machine learning, T cell signaling

## Abstract

Chimeric antigen receptor (CAR) T cell therapy has transformed the treatment of hematologic malignancies, yet its efficacy in solid tumors and durability across broader application remain limited. A central challenge lies in how CAR signaling is initiated, amplified, and regulated over time. Unlike the native T cell receptor (TCR), CARs are synthetic, modular receptors whose signaling output is dictated by the composition and spatial organization of their extracellular, transmembrane, and intracellular domains. Emerging evidence suggests that CAR signaling requirements are not static: insufficient signaling at early time points can impair activation and tumor clearance, whereas excessive or prolonged signaling promotes exhaustion, toxicity, and loss of persistence. More recent CAR designs therefore emphasize fine-tuned signaling, embracing a “less-is-more” paradigm to balance potency with durability. In this review, we summarized recent advances in CAR signaling biology, focusing on temporal signaling thresholds, modular design principles, and emerging strategies to precisely control signal strength and quality. Finally, we discuss how high-throughput screening, computational modeling, and machine learning approaches may enable disease-specific, personalized CAR designs in the future.

## Introduction

Chimeric antigen receptor (CAR) T cell therapy is a clinically validated strategy for redirecting cytotoxic T lymphocytes against malignant cells through synthetic receptor engineering (1–5). By fusing single-chain variable fragments (scFvs) to intracellular T cell signaling modules, CARs confer MHC-unrestricted antigen specificity and drive potent antitumor responses even in heavily pretreated patients (6, 7). However, durable remissions remain largely confined to a subset of hematologic malignancies, and extension to solid tumors and other diseases has proven challenging (8–10).

Antigen selection, T cell trafficking, and the tumor microenvironment (11) are commonly cited barriers, but all three converge on a deeper issue, which is the intrinsic signaling architecture of CARs (12, 13). Early CAR designs assumed that coupling antibody-based recognition to canonical T cell signaling domains would recapitulate native T cell receptor (TCR) function (14–17), an assumption that underestimated the complexity of TCR signaling, which is evolutionarily optimized for sensitivity, adaptability, and self-regulation (18). CARs are synthetic constructs assembled from modular components that lack most of the regulatory features of the native TCR. Their signaling often deviates from physiological norms in magnitude, duration, and pathway selectivity. In some settings, CARs fail to generate enough early signal to drive activation; in others, they trigger chronic overstimulation that accelerates exhaustion, cytokine release syndrome (CRS), and loss of persistence (19, 20). Both extremes are clinically detrimental.

**Figure 1:**
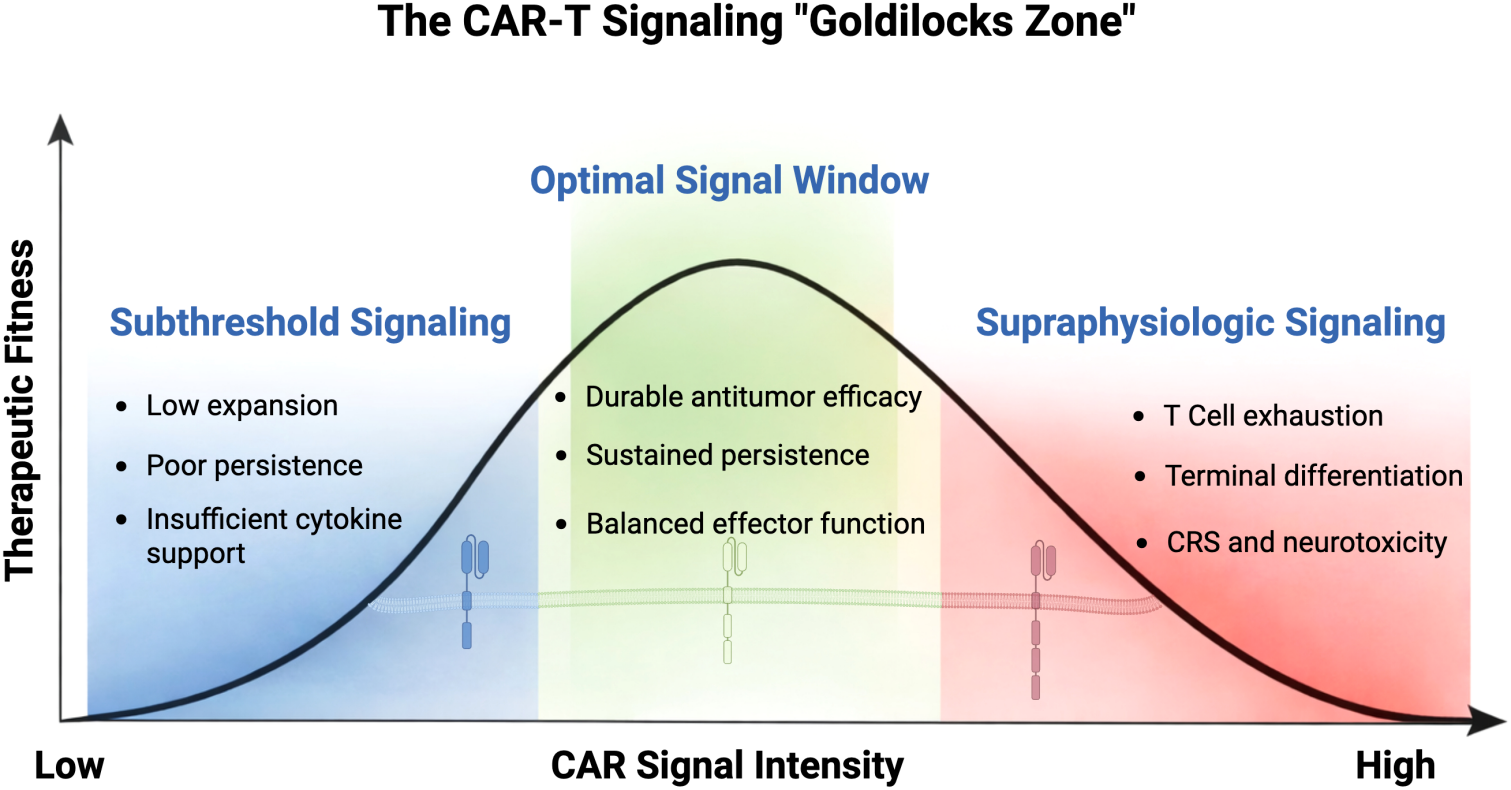
The CAR-T signaling ‘Goldilocks Zone’. Schematic illustrating the relationship between CAR signal intensity and therapeutic fitness. CAR-T cell function is governed by a nonlinear, bell-shaped response curve in which both insufficient and excessive signaling impair efficacy. At low signal intensity (subthreshold signaling), CAR-T cells exhibit limited expansion, poor persistence, and inadequate cytokine support, resulting in insufficient antitumor activity. In contrast, high signal intensity (supraphysiologic signaling) drives T cell exhaustion, terminal differentiation, and increased risk of cytokine release syndrome (CRS) and neurotoxicity. This model highlights the need for precise tuning of CAR signaling strength to maximize therapeutic benefit while minimizing toxicity, providing a conceptual framework for rational CAR design. Only an optimal signaling window achieves durable antitumor efficacy, sustained persistence, and balanced effector function.

A key conceptual advance in the field is the recognition that CAR signaling requirements are temporally dynamic. Robust early signaling is often essential to overcome low antigen density, stromal exclusion, and immunosuppressive cytokines, but once engagement and expansion have occurred, sustained high intensity signaling becomes maladaptive and drives terminal differentiation, exhaustion, metabolic dysfunction, and inflammatory toxicities (21). These opposing requirements support a temporal model in which initiation, amplification, and maintenance each demand different signaling properties (**Figure 1**). Rational CAR design must therefore consider not only what a receptor signals, but when and how much.

This review summarizes recent advances in CAR signaling through this temporal lens. We first contrast CAR and TCR signaling to highlight intrinsic limitations of synthetic receptors. We then examine strategies for enhancing signaling (“the more, the better”), followed by emerging approaches that fine-tune to preserve long-term fitness (“less is more”). Finally, we explore how CAR modularity, combined with high-throughput screening and machine learning, is enabling a shift from empirical optimization to rational, disease-specific design.

## 2 Fundamentals of CAR signaling and architecture compared with the TCR

CARs are developed to give T cells antibody-like specificity while preserving cytotoxic function (6, 22, 23). Unlike conventional TCRs, which recognize peptide-MHC complexes, CARs engage surface antigens directly and thus bypass tumor immune evasion via MHC downregulation (13). The canonical CAR consists of four modular components: an extracellular antigen-recognition domain, typically an scFv, a hinge or spacer that sets synapse geometry, a transmembrane anchor, and an intracellular signaling domain for T cell activation. On antigen engagement, CAR clustering triggers phosphorylation cascades that recapitulate key elements of TCR signaling, driving cytotoxicity, cytokine production, proliferation, and differentiation (24).

The native TCR is a highly regulated multi-subunit complex carrying ten immunoreceptor tyrosine-based activation motifs (ITAMs) across CD3 chains, enabling sensitive, tunable responses to low antigen levels (25). In contrast, most CARs rely on CD3ζ with only three ITAMs, reducing signaling complexity and regulatory control (6, 18). CARs also display altered synapse organization, reduced sensitivity to low antigen density, and a tendency toward tonic signaling from clustering or ligand-independent activation, particularly in tumors with chronic antigen stimulation, underlies CAR-T exhaustion and toxicity and motivates the engineering of signal amplitude and duration (19, 26, 27).

**Figure 2:**
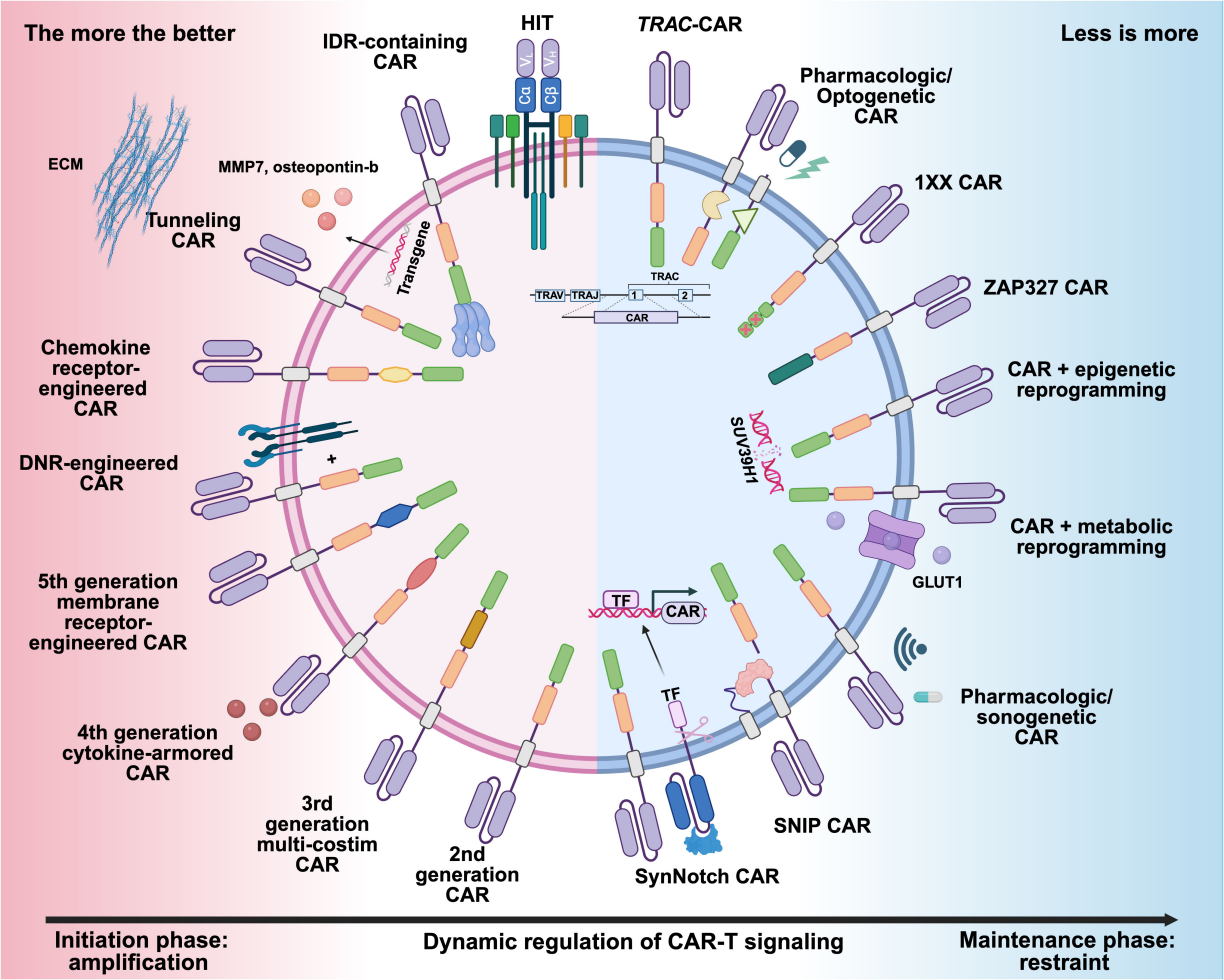
Temporal and modular regulation of CAR-T signaling. Schematic illustrating CAR-T engineering strategies across the signaling continuum. ‘The more the better’ approaches enhance activation through costimulation, cytokine armoring, receptor clustering and improved trafficking and tumor infiltration. ‘Less is more’ strategies focus on limiting excessive signaling to preserve persistence, including TRAC targeting, signaling-tuned constructs (e.g., 1XX, ZAP327), and transcriptional, epigenetic, and metabolic reprogramming. Dynamic control systems such as SynNotch, SNIP, and pharmacologic, optogenetic, or sonogenetic CARs enable reversible regulation of CAR activity. HIT (HLA-independent T cell receptor): antibody-based receptor assembled with the endogenous TCR–CD3 complex but integrated into the endogenous TRAC locus, where expression is regulated by the native TRAC promoter and TCR regulatory elements.

First-generation CARs, described in the late 1980s and early 1990s, fused antibody-derived binding domains directly to CD3ζ (22, 23). They mediated antigen-specific cytotoxicity *in vitro* but showed limited *in vivo* efficacy (16, 17). CD3ζ alone could not sustain expansion, survival, and memory formation. This signal insufficiency drove the next generations of CAR design, initially under the assumption that stronger signaling would yield stronger antitumor activity (**Figure 2**).

## 3 The more the better: enhancing CAR signaling

The weak persistence and efficacy of first-generation CARs led to the addition of costimulatory domains, reflecting the physiological requirement for signal 2 in T cell activation (16, 28–30). Second-generation CARs, which fuse CD3ζ with a single costimulatory domain, most often CD28 or 4-1BB (CD137), represent a major breakthrough (31). CD28 promotes rapid effector differentiation and glycolytic metabolism, while 4-1BB enhances mitochondrial fitness and long-term persistence (32). As a result, second-generation CARs significantly improve T cell proliferation, survival, and clinical efficacy, particularly in CD19-targeted therapies for B cell malignancies (2, 3). Third-generation CARs add a second costimulatory domain (e.g., CD28 plus 4-1BB or OX40) to combine rapid effector activity with long-term persistence (33–35), but they have not consistently outperformed the second-generation CARs in the clinic since the extra signal has often produced more severe CRS and T cell dysfunction (36, 37). Fourth- and fifth- generation CARs therefore incorporate additional regulatory elements such as cytokines, chemokines, and synthetic signaling modules to improve CAR functions.

### 3.1 Cytokine armoring and cytokine receptor engineering

A complementary strategy to enhance CAR-T function is to engineer CAR-T cells to express cytokines or their corresponding receptors, thereby reinforcing activation through autocrine and paracrine loops. Fourth-generation CARs secreting cytokines upon CAR signaling are known as TRUCKs (T cells redirected for universal cytokine-mediated killing). Unlike receptor-intrinsic modifications, which act only upon antigen engagement, cytokine-based strategies can sustain T cell survival, proliferation, and effector function even in antigen-sparse or immunosuppressive environments. These strategies fall into four broad classes: homeostatic cytokines that support survival and memory, pro-inflammatory cytokines that amplify effector functions and reshape the TME, immunomodulatory cytokines that tune differentiation and persistence, and engineered receptors that co-opt or rewire TME cytokine signals (38–59).

#### 3.1.1 Homeostatic cytokines

IL-7 and IL-15 are γ-chain cytokines that promote T cell antigen-independent proliferation, metabolic fitness, and survival, making them useful for preventing CAR-T senescence. Markley and Sadelain showed that IL-7 and IL-21 outperform IL-2 and IL-15 in sustaining durable antitumor immunity in xenograft models, with IL-7 driving iterative expansion and IL-21 promoting long-lived effector memory (56). These findings motivated transgenic co-expression strategies that provide an autocrine survival loop without exogenous cytokine administration.

The 7x19 CAR (IL-7 plus CCL19) was the first systematic demonstration that a single vector encoding a cytokine and a homeostatic chemokine could reconstitute T cell-supportive zones within tumors, driving complete regression of established solid tumors and promoting dendritic cell and endogenous T cell infiltration (51). The 7×21 (IL-7 and CCL21) variation achieved superior tumor control, anti-angiogenic activity, and efficacy against antigen-heterogeneous tumors without lymphodepletion (48). IL-7 has also been combined with a dual-antigen binary CAR targeting PSCA and MUC1 to address antigen escape in solid tumors (39). In the clinic, GD2-directed CAR-T cells carrying a constitutively active IL-7 receptor (C7R) showed encouraging early responses and transient neurological improvement in patients with pediatric CNS tumors (58). In parallel, C7R-armored AXL-CAR-T cells enhanced CAR-T proliferation, cytotoxicity, and *in vivo* persistence in triple-negative breast cancer models (47).

IL-15 preferentially expands CD8+ effector memory and NK cells sustains mitochondrial fitness without the rapid exhaustion seen with IL-2. Co-expression of IL-15 and IL-21 in GPC3-CAR-T cells for hepatocellular carcinoma gave the best expansion and *in vivo* persistence of any cytokine combination tested, mechanistically linked to sustained TCF-1 expression and enrichment of stem-cell memory populations (41, 49). Co-expression of the full IL-15/IL-15Rα transpresentation complex on NKG2D-CAR-T cells further activated JAK/STAT5 pathway and enhanced cytotoxicity in pancreatic cancer models (43). Clinically, Steffin and colleagues reported a 66% disease control rate and 33% objective response rate in patients with solid cancers treated with IL-15-armored GPC3 CAR-T cells, with responders showing SWI/SNF epigenetic repression and upregulation of FOS/JUN family members in tumor-infiltrating cells (41). IL-15 armoring was also explored in combination with a tumor-localized anti-PD-L1 fusion protein (αPD-L1-IL-15), though this combination proved inferior to αPD-L1-IL-12 in both safety and efficacy in preclinical prostate and ovarian cancer models (42).

#### 3.1.2 Pro-inflammatory cytokines

IL-12 drives Th1 polarization, IFN-γ production, and macrophage activation. Constitutive IL-12 secretion by MUC16ecto-targeted CAR-T cells depleted tumor-associated macrophages, resisted PD-L1-mediated suppression, and improved survival in ovarian cancer xenograft models (54), and this CAR-T has been clinically evaluated in heavily pre-treated patients with high-grade ovarian cancer, though the best responses seen were stable disease (60). Inducible IL-12 release combined with CAR-T recruited macrophages to eliminate antigen-loss variants, directly addressing antigen escape (55). IL-12-secreting CAR-T cells co-expressing CCR5 combined the CCL4/5-CCR5 mediated cell trafficking with macrophage reprogramming to overcome immunosuppression in esophageal carcinoma models (40). To mitigate systemic toxicity associated with IL-12, Murad and colleagues engineered CAR-Ts that secrete a bifunctional αPD-L1-IL-12 fusion protein to concentrate cytokine activity at PD-L1-expressing tumor cells, which improved T cell trafficking and localized IFN-γ production with far less systemic inflammation (42). A complementary CRISPR-based strategy leveraged tumor-restricted endogenous promoters (NR4A2 and RGS16) to drive IL-12 and IL-2 expression exclusively at the tumor site, giving enhanced polyfunctionality with favorable safety (61).

IL-18 acts as a T cell growth factor as well as a polarizing signal that converts CAR-T cells toward a T-bet^high^ FoxO1^low^ effector state that is particularly active in solid tumors. Hu and colleagues first showed that IL-18-secreting CAR-Ts proliferate in a TCR- and IL-18R-dependent manner and augment antitumor activity against melanoma in immunocompetent model (52). IL-18 armoring has also been explored in inducible systems, with NFAT-promoter-driven IL-18 expression integrated into an all-in-one lentiviral TRUCK vector producing GD2-CAR-Ts that activate cytokine release only upon tumor contact (62). In addition, CARs with inducible IL-18 release repolarized the TME macrophage landscape toward M1 phenotypes, depleted Tregs and suppressive DCs, and eradicated pancreatic and lung tumors refractory to conventional CAR-T therapy (53). IL-18 armored DLL3-targeted CAR-Ts preserved memory phenotype, reduced exhaustion, and produced durable responses in multiple small cell lung cancer models (45). This effect was further enhanced by PD-1 blockade, suggesting productive synergy between cytokine armoring and checkpoint inhibition. These advances converged clinically. Svoboda and colleagues reported that IL-18-secreting huCART19-IL18 cells achieved an 81% overall and 52% complete response rate in lymphoma patients relapsed after prior CD19 CAR-T therapy, demonstrating that IL-18-driven amplification can rescue refractory disease at low doses (59).

IL-36γ is the most recently characterized pro-inflammatory armoring cytokine. Zuo and colleagues demonstrated that IL-36γ-armored CAR-T cells not only enhanced direct cytotoxicity but also reprogrammed tumor-infiltrating neutrophils into tumoricidal, antigen-cross-presenting cells, eliciting endogenous T cell responses against antigens beyond the CAR target (38). The resulting epitope spreading was sufficient to reject antigen-negative tumor rechallenge, extending cytokine armoring from T cell-intrinsic amplification to engagement of the broader cancer immunity.

#### 3.1.3 Immunomodulatory cytokines

CAR-T cells secreting an engineered IL-2 superkine (Super2) together with IL-33 effectively controlled tumor growth in multiple solid tumor models by recruiting and activating a broad repertoire of endogenous innate and adaptive immune cells, showing that cytokine armoring can act systemically on NK cells, tumor-specific T cells, and innate immune cells to compensate for loss of individual effectors and thus overcome tumor escape (46).

IL-23 acts more selectively. Ma and colleagues found that TCR stimulation upregulates the IL-23 receptor and the IL-23α p19 subunit but not the p40 subunit (57). Transgenic p40 expression therefore resulted in selective proliferation in T cells via autocrine IL-23 signaling. More importantly, p40-subunit engineered CAR-T (p40-Td CAR) outperformed IL-15- and IL-18-armored CARs preclinically with a more favorable toxicity profile.

In addition, IL-10-expressing CAR-T cells were shown to resist dysfunction and mediate durable clearance of solid tumors and metastases by reducing exhaustion-associated programs, preserving stem-like characteristics, and maintaining effector function during chronic antigen exposure (63).

#### 3.1.4 Cytokine receptor engineering

In addition to the cytokines, the cytokine receptors can also be engineered into CARs to redirect immunosuppressive TME signals into T cell-activating inputs. The inverted cytokine receptor (ICR) concept exemplified this principle by fusing the IL-4 extracellular domain to the IL-7 receptor intracellular domain (4/7 ICR), converting immunosuppressive IL-4 into an IL-7-like survival signal (64). Paired with a second-generation CAR, this delivered all three physiological activation signals (TCR engagement, costimulation, and cytokine support) needed for durable control in IL-4-rich breast cancer models. A 4/21 ICR variant activated the STAT3 pathway and promoted Th17-like polarization and cytotoxicity in IL-4-positive tumors (65), and a TGF-β/IL-7 inverted receptor converted the most prevalent immunosuppressive TME cytokine into an IL-7 survival signal in PSMA-CAR-Ts for prostate cancer (66). To bypass the pleiotropic and potentially toxic effects of wild-type IL-2 on regulatory T cells, Sockolosky and colleagues engineered an orthogonal IL-2/IL-2Rβ pair that signals only with itself; introducing the orthogonal receptor into CAR-Ts allowed selective *in vivo* expansion of transduced cells with negligible off-target effects (50).

Dominant-negative receptors convert inhibitory signals into amplifying and activating signals. Co-expression of a dominant-negative TGF-βRII in PSMA-CAR-T cells conferred TGF-β resistance, enhanced proliferation and persistence, and eradicated prostate tumors in aggressive preclinical models (67). A phase I trial of PSMA-targeting TGF-β-insensitive armored CAR-T in patients with metastatic castration-resistant prostate cancer confirmed feasibility and safety and produced PSA reductions, though failure was accompanied by upregulation of additional TME-localized inhibitory molecules, pointing to the need for combinatorial strategies (68). A bispecific IL-13Rα2/TGF-β CAR that actively converted TGF-β from immunosuppressant to immunostimulant by treating it as a second CAR antigen demonstrated improved T cell infiltration and survival in glioblastoma models (69). CARs armored with dominant-negative receptors were also validated clinically in Hodgkin lymphoma, where DNRII-expressing LMP-specific T cells expanded up to 100-fold, persisted over four years, and induced complete responses in patients who had failed prior unmodified T cell therapy (70).

### 3.2 Trafficking and microenvironmental signal enhancement

Early signaling is also constrained by physical access to tumor cells, and enhancing T cell infiltration indirectly boosts effective signaling by raising the frequency of productive CAR-T-tumor encounters. CAR-T cells engineered to overexpress CXCR3 variants exploited CXCL10-rich tumor milieu to improve infiltration and tumor control in solid tumors (71, 72). Similarly, CARs with CCR5 overexpression and IL-12 secretion showed enhanced trafficking and reprogrammed macrophage-mediated immunosuppression, resulting in improved antitumor efficacy (40). Van Pelt and colleagues addressed extracellular matrix barriers by overexpressing MMP-7 or osteopontin-b in GD2-CAR-Ts; these “tunneling CARs” exhibited improved extravasation and interstitial migration in ECM-dense tumors, translating into superior tumor control (73). Related strategies include secretion of VEGF-blocking scFvs (74), which neutralize VEGF-mediated immunosuppression and enhance CAR-T intratumoral activation, and IL-7-armed binary CARs (39), which support local expansion and persistence while maintaining robust early effector responses.

### 3.3 Engineering proximal signal amplification

Recent studies have directly addressed CAR hypo-responsiveness to low antigen density. Rotiroti and colleagues co-expressed CARs with a membrane-tethered version of SLP-76 (MT-SLP-76), a key adaptor protein in TCR signaling (75). Pre-positioning SLP-76 at the membrane improved recruitment of ITK and PLCγ1, lowered the activation threshold, and restored killing of antigen-low tumors. This work demonstrated that engineered recruitment of proximal signaling components can compensate for structural limitations of CARs. In parallel, Zhang and colleagues introduced intrinsically disordered regions (IDRs) into CAR constructs to promote biomolecular condensation at the immune synapse (76). IDR-containing CARs formed more robust receptor clusters and membrane-proximal phosphorylation, improving cytotoxicity against low-antigen cancers without spontaneous activation in the absence of antigen. These findings highlight the importance of biophysical organization in signal amplification and suggest that CAR performance can be improved by mimicking phase-separated microcluster dynamics of TCR signaling.

Shi and colleagues took a different track by integrating pre-T cell receptor pTα 1A domain into CD28-based CARs to enhance mRNA translation via sustained phosphorylation of YBX1 (77). pTα-containing CARs exhibited greater expansion, reduced exhaustion, and improved tumor control in both hematologic and solid tumor models. Together, these studies show that insufficient early signaling can be overcome by strengthening receptor clustering, adaptor recruitment, and biosynthetic coupling to reprogram CARs to respond more effectively during tumor engagement.

An alternative approach to amplifying early CAR signaling is to reconstitute antibody-mediated recognition within the native TCR signaling machinery. Mansilla-Soto and colleagues developed HLA-independent T cell receptors (HIT receptors), in which antibody-derived variable domains are integrated into the endogenous TCR complex through TRAC locus engineering, enabling antigen recognition through the full TCR–CD3 signaling apparatus rather than a synthetic CD28/4-1BB/CD3ζ receptor (78). Compared with conventional CARs, HIT receptors exhibited substantially greater antigen sensitivity and maintained tumor recognition at antigen densities below the activation threshold of even highly optimized CD28-based CARs. This enhanced sensitivity translated into superior control of antigen-low leukemia and solid tumor models, highlighting the importance of physiological signal amplification mediated by the endogenous TCR-CD3 complex (79).

### 3.4 Expanding the amplification toolkit

Beyond the specific examples above, a wide range of co-engineering strategies have been explored to enhance early CAR signaling and function. These include co-expression of chemokine receptors (CCR2b, CXCR1, CXCR2), anti-apoptotic molecules (BCL2L1), adaptor modules (SLP76 variants), cytokine combinations such as IL-15/IL-21 or IL-7/CCL19, PD-1 blockade modules, MyD88/CD40 signaling switches, TLR2 domains, and enzymatic modifiers such as heparanase (HPSE) for tumor penetration (75, 80–91). Each reflects the same principle: when early activation is inadequate, augmenting signaling, survival, or tumor infiltration can restore potency. However, unrestrained amplification carries long-term costs, motivating the alternative strategies discussed next.

## 4 Less is more: fine-tuning signaling for persistence

The same signaling intensity that drives early rapid tumor clearance after infusion maladaptive when sustained. Chronic antigen engagement enforces T cell exhaustion, which is a transcriptionally and epigenetically stabilized cell state characterized by progressive loss of proliferative capacity, attenuated cytokine polyfunctionality, metabolic dysfunction, and constitutive upregulation of inhibitory receptors including PD-1, TIM-3, and LAG-3 (92). Unlike acute infections, in which antigen clearance triggers contraction and memory formation, tumors provide persistent antigens that continually re-engage the CAR. Because CARs couple activation and costimulatory signals into a single invariant molecule, they cannot replicate the temporal separation of these cues seen in physiological TCR activation and therefore deliver prolonged downstream stimulation that accelerates terminal differentiation. The costs extend beyond individual cells: depletion of the stem-like progenitor pool forecloses durable responses, while excessive cytokine secretion drives CRS and inflammatory toxicity, narrowing the therapeutic window (93). An additional and mechanistically distinct contributor is tonic signaling, which is a ligand-independent, constitutive CAR activation arising from antigen-independent scFv clustering, imposing a baseline phosphorylation burden that accelerates differentiation even before the first tumor encounter, and whose magnitude correlates directly with the density of positively charged patches (PCPs) on the scFv surface (19, 94). Therefore, restraining CAR signaling is not a compromise of efficacy but a prerequisite for durable clinical benefit (**Figure 2**).

### 4.1 Structural and proximal signaling attenuation: from receptor geometry to signal calibration

The most direct way to reduce CAR signal amplitude is to modify the receptor itself to dampen activation. At the receptor-ligand interface, lowering scFv affinity shortens bond dwell times and reduces cumulative phosphorylation per encounter while preserving serial killing. Ghorashian and colleagues provided the first clinical proof with the CAT low-affinity CD19 CAR in pediatric ALL: 12 of 14 patients achieved remission with improved expansion and persistence relative to high-affinity historical controls, and with near-zero severe CRS (95). Low-affinity CARs also reduce trogocytosis that transfers target antigen from tumor cell to T cell membranes during prolonged receptor engagement, preserving antigen on tumor cells and limiting trogocytosis-driven activation-induced cell death (96). Affinity fine-tuning of CAIX-targeting CARs further widened the therapeutic window between antigen-high tumor cells and antigen-low normal tissues, addressing on-target/off-tumor toxicity (97).

Tonic signaling can also be reduced by addressing the clustering forces that drive it. Chen and colleagues identified PCPs on the scFv surface as the primary driver of antigen-independent CAR aggregation. Reducing PCP alleviated exhaustion in high-tonic GD2 and CSPG4 CARs, while adding PCP to the low-tonic CD19 CAR improved *in vivo* persistence, establishing PCP as a designable parameter for optimizing steady-state signaling tone (94, 98). At the genomic level, CRISPR/Cas9-mediated insertion of the CAR into the TRAC locus places expression under endogenous TCRα regulatory control, allowing physiological expression levels and internalization and re-expression of the receptor following antigen encounter. TRAC-targeted CAR-T cells markedly outperformed randomly integrated counterparts in ALL models, with delayed exhaustion, preserved stemness, and superior tumor rejection (99). Weaker promoters and lower CAR surface density reinforce this principle: the MND promoter-driven low-density CAR produced milder IFN-γ responses with equivalent remission rates compared to high-density EF1α-driven CARs, while BCMA-CAR-high cells independently predicted worse outcomes than CAR-low products (100, 101). Hinge domain rigidity provides another structural lever: deleting two glycine residues in the CD8α hinge lowered proinflammatory cytokine secretion, and extended *in vivo* survival under high tumor burden without compromising cytotoxicity (102). Similarly, optimization in the length of hinge and transmembrane resulted safe and potent anti-tumor responses (103). Reconstitution of a TCR-like architecture through STAR receptors restores physiological signaling organization, reducing tonic signaling while enhancing persistence and long-term function (104–106).

Intracellularly, reducing the number of active CD3ζ ITAMs directly attenuates phosphorylation cascades and lowers peak signaling amplitude. Feucht and colleagues demonstrated that single-ITAM CARs (1XX) biased T cells toward memory rather than effector states, improving persistence without sacrificing early cytotoxicity (107). Majumdar and colleagues showed that individual CD3ζ ITAM are non-redundant: the weakest-signaling ζCCC configuration produced the cells least prone to differentiation, terminal exhaustion, and activation-induced cell death (108). Clinically, the CD19-1XX CAR, a CD28-based construct with a calibrated ITAM module, achieved an 82% overall response rate at a dose of only 25 million cells, with CAR-T persistence beyond one year in ongoing remitters and low rates of severe toxicity (109). Attenuating PI3K signaling within CD28 provides a complementary strategy. The SAVVY CD28 rheostat, a single asparagine-to-phenylalanine substitution that attenuates PI3K/AKT/mTOR engagement, produced superior memory phenotypes and better tumor control in low-antigen-density and PD-L1-high models without sacrificing effector function (110). Finally, at the kinase level, ZAP327, a modified ZAP-70 kinase domain incorporated into the CAR intracellular tail, represents a mechanistically distinct strategy compared with all currently FDA-approved CAR designs. This engineered module promotes expansion of stem-like memory T cell populations, and shifts cellular metabolism toward oxidative phosphorylation, resulting in superior performance in antigen-low tumor models (111). Notably, ZAP327 CAR-T cells produce significantly lower levels of inflammatory cytokines while maintaining comparable cytotoxic activity, suggesting an improved therapeutic benefit with enhanced safety.

### 4.2 Transcriptional, epigenetic, and metabolic reprogramming

Structural restraint at the receptor level is necessary but insufficient when the cell’s own transcriptional and epigenetic machinery is already wired toward exhaustion. A parallel set of strategies intervenes downstream at the level of chromatin, transcription factor, and metabolism.

#### 4.2.1 Transcription factor engineering

c-Jun is functionally deficient in exhausted CAR-T cells, opening chromatin at AP-1 exhaustion-associated loci while closing stemness loci. Lynn and colleagues showed that c-Jun overexpression restored IL-2 production, reversed exhaustion-associated chromatin remodeling, and produced cells with enhanced expansion and less terminally differentiated phenotype across five *in vivo* tumor models (112). TCF-1 (TCF7), the master regulator of T cell stemness and progenitor exhausted states, is typically low in manufactured CAR-T products. Forced TCF-1 expression generated cells enriched in naive-like and stem-like subsets with reduced apoptosis and sustained cytotoxicity (113). T-bet overexpression offers a distinct transcriptional intervention that enhances effector sensitivity rather than restraining exhaustion; by enforcing Th1 polarization in CD4+ cells and improving polyfunctional cytokine production, T-bet improved response to low-CD19-density leukemia without compromising persistence or secondary responses to tumor rechallenge (114).

#### 4.2.2 Epigenetic reprogramming

Exhaustion is stabilized by epigenetic silencing, primarily through H3K9 trimethylation at effector and stemness loci. Two complementary studies identified the H3K9 trimethyltransferase SUV39H1 as a tractable target. CRISPR disruption of SUV39H1 in CD28-based CAR-T cells enhanced early expansion, long-term persistence, and antitumor efficacy in leukemia and prostate cancer models, with persisting cells maintaining accessible memory transcription factor chromatin and reduced inhibitory receptor expression upon multiple rechallenges (115). Similarly, SUV39H1 ablation in 4-1BB-based CAR-T cells reprogrammed tumor-infiltrating populations toward self-renewing stem-like states, protecting mice from solid tumor relapse and rechallenges for months (116). These parallel findings established SUV39H1-mediated H3K9 methylation as a shared epigenetic brake on CAR-T cell longevity.

Exhaustion is not epigenetically fixed: transient CAR signaling cessation reversed exhaustion-associated chromatin patterns, restored antitumor function, and globally remodeled transcriptome toward memory-like states (117). Vitamin C supplementation during manufacturing enforced a CD45RA+CD62L+ stem cell memory phenotype through TET-mediated DNA demethylation, enhancing serial killing and *in vivo* tumor control (118). An all-RNA CRISPRoff/CRISPRon platform enabled stable, multiplexed epigenetic silencing or activation of endogenous genes without double-strand DNA breaks; combined with TRAC locus CAR knock-in, it improved CAR-T-mediated tumor control preclinically, demonstrating that integrated genetic and epigenetic programming can cooperatively optimize CAR-T function (119).

#### 4.2.3 Metabolic fitness

Signaling intensity and metabolic states are bidirectionally coupled: chronic signaling drives glycolytic dependence and mitochondrial fragmentation, while mitochondrial fitness supports the oxidative phosphorylation characteristic of memory T cells. GLUT1 overexpression in CAR-T cells produced broad metabolic reprogramming, resulting in increased glycolytic reserve and oxidative phosphorylation and enhanced glutathione-mediated ROS resistance. These effects are subsequently associated with decreased exhaustion, increased Th17 differentiation, and superior *in vivo* persistence (120). Nitric oxide (NO) accumulation is a distinct metabolic threat: continuous CAR engagement drives excessive NO production and suppresses S-nitrosoglutathione reductase (GSNOR), causing protein S-nitrosylation, impaired mitochondrial dynamics, and loss of stemness. Enforced GSNOR expression reversed this cascade, restoring mitochondrial integrity, promoting memory differentiation, and enhancing antitumor expansion in chronic antigen stimulation (121).

### 4.3 Temporal and pharmacologic control: engineered circuits and small-molecule restraint

Structural and epigenetic restraint encode “less is more” permanently into the CAR-T product. Approaches that implement restraint dynamically to allow high signaling in the early stage and reduced signaling in the later stage are clinically attractive.

#### 4.3.1 Pharmacologic, photogenetic, and ultrasonic on/off switches

Externally controllable CAR-T systems provide a powerful strategy for dynamic regulation of T cell activity, enabling precise control over signaling intensity, timing, and location. One of the earliest approaches involves small molecule–gated “ON-switch” CARs, in which antigen recognition and intracellular signaling domains assemble only in the presence of a heterodimerizing drug, allowing reversible and titratable activation without compromising antigen specificity (122). Optogenetic systems further extend this concept by enabling spatiotemporal control of CAR signaling using light. Light-inducible platforms such as LINTAD allow non-invasive activation of CAR-T cells with high precision, while nano-optogenetic approaches (LiCAR) incorporate upconversion nanoparticles to achieve deep-tissue activation, overcoming limitations in light penetration (123, 124). More recently, sonogenetic systems have enabled ultrasound-controlled CAR expression, in which focused ultrasound (FUS) activates CAR signaling in a spatially confined and sustained manner, providing a clinically translatable approach for deep-tissue tumors (125, 126). In parallel, pharmacologic strategies offer reversible control of CAR signaling pathways. Dasatinib acts as a rapid and potent off-switch by inhibiting LCK and downstream CD3ζ/ZAP-70 phosphorylation, inducing a transient “rest” state that mitigates cytokine release syndrome while preserving subsequent antitumor function (117, 127). Similarly, ibrutinib improves CAR-T fitness by reducing exhaustion-associated signaling and enhancing expansion in both preclinical and clinical settings (128). Collectively, these approaches transform CAR-T therapy from a constitutively active system into a programmable platform with tunable signaling, offering a promising path toward improved safety and precision.

#### 4.3.2 Protease-regulated and degron-tagged CARs

Labanieh and colleagues developed SNIP CARs, in which the CAR-T activity is regulated by an FDA-approved small molecule drug. SNIP CARs demonstrated full effector function with the drug and no leaky activity in the absence of it. Critically, drug withdrawal following toxicity onset rescued animals in a CAR lethality model, and reduced drug dosing opened a therapeutic window that enabled tumor eradication without toxicity (129).

The CTLA-4 cytoplasmic tail (CCT) fusion provides an autonomous built-in attenuation mechanism without exogenous drugs. Fusing CCT to the CAR C-terminus installs a constitutive endocytosis/recycling/degradation cycle that progressively reduces surface CAR density at steady-state. CCT-fused CARs exhibited reduced trogocytosis, lower tonic signaling, stronger central memory phenotypes, increased persistence, and superior antitumor efficacy in relapsed leukemia models (130).

#### 4.3.3 Synthetic receptor circuits for autonomous temporal control

SynNotch receptors, introduced by Morsut and colleagues as a general platform for contact-triggered gene regulation, allow CAR expression to be restricted to the tumor site and initiated only after a defined priming signal (131). AND-gate synNotch-CAR circuits arm T cells only in the presence of combinatorial tumor antigens, sparing single-antigen bystander tissues *in vivo* (132). In solid tumors, synNotch-gated CAR expression prevented the tonic signaling and exhaustion that accompany constitutive constructs, yielding superior tumor control and long-lived non-exhausted T cells in mesothelioma and ovarian cancer models (133). In glioblastoma, EGFRvIII or MOG synNotch priming circuits restricted CAR activity to the brain, giving superior PDX tumor control without off-tumor toxicity (134). The fully humanized SNIPR platform extended these principles with tunable sensing and dosed payload delivery suitable for clinical translation (135). Drug-inducible costimulatory systems, such as the inducible MyD88/CD40 module controlled by a chemical inducer of dimerization, provide a clinician-operated variant, allowing strong costimulation only when the small-molecule activator is administered (88).

The most effective “less is more” designs integrate restraint across multiple temporal and molecular layers: structural attenuation of tonic and peak signaling (low-affinity scFv, PCP reduction, ITAM calibration, TRAC knock-in) combined with transcriptional and epigenetic enforcement of stemness (c-Jun, TCF-1, SUV39H1 editing), metabolic support (GSNOR, GLUT1), pharmacologic or circuit-based temporal control (dasatinib, SNIP CARs, synNotch). The “less is more” and “more is better” strategies are opposite but sequential with amplification strategies serve the initiation phase and restraint strategies serve the maintenance phase. The ideal CAR-T product should encode both, either through a self-regulated synthetic circuit or through a rational combination of complementary modifications that together implement the full staged signaling model.

## 5 Artificial intelligence-accelerated design of smart CAR-T cells

**Figure 3:**
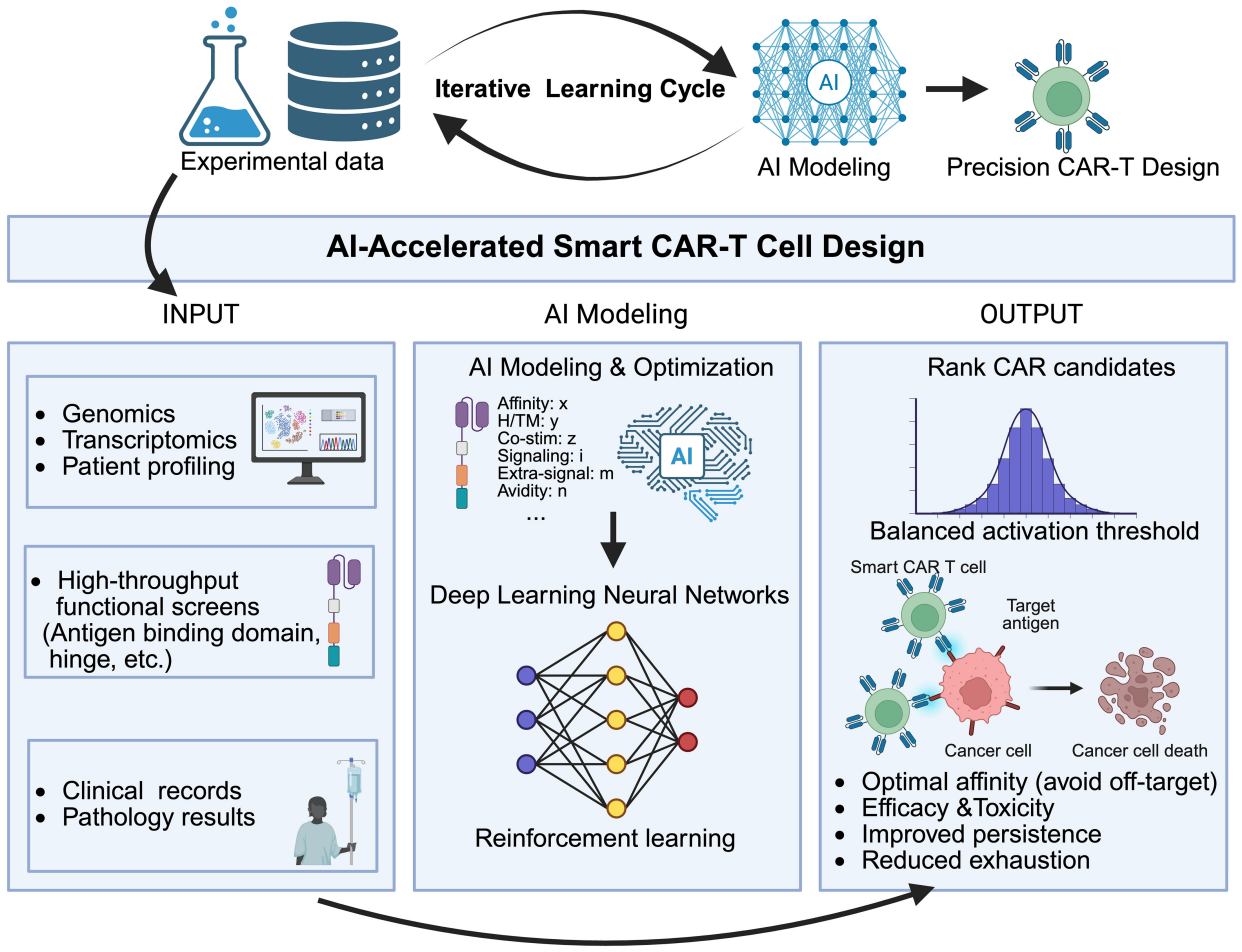
AI-accelerated design of smart CAR-T cells. Multi-modal inputs, including genomics, transcriptomics, high-throughput functional screens, and clinical data, are integrated into AI models to learn relationships between CAR design parameters and functional outcomes. Machine learning approaches, including deep learning and reinforcement learning, model nonlinear interactions and optimize CAR features such as affinity, signaling strength, and costimulation. The output is the ranking and selection of optimized CAR constructs with balanced activation thresholds, improved efficacy, enhanced persistence, and reduced exhaustion, enabling precision CAR-T design.

The rapid evolution of CAR engineering has expanded the design space of CAR-T cells far beyond conventional constructs composed of a single antigen-binding domain and canonical costimulatory motifs. Advances in modular receptor design and high-throughput screening now permit systematic exploration of diverse CAR architectures, revealing that CAR-T function emerges from complex, nonlinear interactions among multiple parameters (136, 137). In this context, artificial intelligence (AI) has emerged as a powerful framework to model such interactions, extract design principles from large-scale datasets, and guide the rational development of next-generation “smart” CAR-T cells with improved efficacy, persistence, and safety (**Figure 3**).

Importantly, AI-driven CAR design should not be viewed as a single computational approach. In the CAR-T context, it may include mechanistic models that simulate receptor–antigen engagement and downstream signaling kinetics; supervised machine-learning models that map CAR structural features to functional outputs; structure-aware deep learning models that optimize antigen-recognition domains; single-cell and multi-omics models that define T-cell activation, memory, and exhaustion states; and iterative optimization frameworks such as Bayesian optimization, active learning, and reinforcement learning (138–146). These model classes differ substantially in their biological assumptions, data requirements, interpretability, and practical outputs. Therefore, distinguishing among them is essential for understanding how computational approaches can be applied to rational CAR-T design (138–140).

### 5.1 AI modeling of CAR activation landscapes and design parameters

CAR-T cell activation does not depend on any single variable but instead arises from the integration of multiple biophysical and signaling parameters, including antigen-binding affinity, CAR surface density, tumor antigen density, and intracellular signaling architecture (137, 147–149). Together, these features define a multidimensional activation landscape that governs T-cell cytotoxicity, proliferation, persistence, and susceptibility to exhaustion.

A central principle emerging from both CAR and T-cell receptor (TCR) biology is the existence of activation thresholds. In TCR signaling, ligand affinity and coreceptor engagement determine whether cytotoxic T lymphocytes are activated (150, 151), remain inert, or enter dysfunctional states when signaling is excessive (152, 153). Analogous behaviors are observed in CAR-T cells, where antigen-binding affinity must be precisely tuned: overly high affinity increases the risk of off-tumor toxicity by enabling recognition of low-antigen–expressing normal tissues, whereas insufficient affinity can lead to failure of activation within heterogeneous tumors.

Receptor expression level represents an additional critical determinant of CAR signaling. Clinical and preclinical studies have shown that CAR surface density correlates with antitumor efficacy (154), but excessive CAR expression can promote tonic signaling, leading to T-cell exhaustion and reduced persistence (19). Promoter choice and vector design further influence CAR expression levels, thereby shaping signaling kinetics and functional outcomes (155). Importantly, CAR activation is also strongly dependent on tumor antigen density, as the probability of receptor engagement increases with antigen abundance (156). These findings collectively highlight that CAR function depends on the interplay between receptor density and antigen density, rather than either parameter alone.

The relationship among these variables is inherently nonlinear. CAR signaling exhibits threshold behavior, saturation effects, and dynamic ranges in which optimal activation occurs. Below threshold, CAR-T cells remain inactive; within an optimal window, they achieve productive effector function; and beyond this range, excessive signaling promotes exhaustion or toxicity (19, 136, 152–154). Capturing such behavior requires analytical frameworks capable of modeling high-dimensional, nonlinear response surfaces. Mechanistic and quantitative models provide one interpretable approach for defining these activation landscapes. Such models can incorporate receptor occupancy, antigen density, CAR surface density, scFv affinity and dissociation rate, receptor clustering, synapse geometry, CD3ζ phosphorylation, ZAP70 recruitment, costimulatory signaling, cytokine production, proliferation, and tumor-killing kinetics (136–139). Their value lies in the ability to estimate a therapeutic activation window in which CAR-T cells respond to antigen-high tumor cells but remain relatively inactive against antigen-low normal tissues. However, these models require experimentally measured parameters and often simplify the full complexity of T-cell signaling, making validation with functional assays essential (138, 139).

Data-driven machine-learning approaches provide a complementary framework by learning relationships directly from CAR-T functional assays and library screens (138–140). By integrating data from CAR-T functional assays, computational models can capture nonlinear interactions among affinity, density, and signaling architecture, enabling prediction of functional outcomes across diverse design conditions. Supervised learning models relate CAR design features to cytokine production, proliferation, and persistence, while regression-based approaches estimate optimal signaling strength (138, 141, 142). Importantly, these models can identify parameter regimes that maximize antitumor activity while minimizing adverse effects, enabling rational CAR design that goes beyond empirical optimization.

In supervised learning frameworks, each CAR construct can be encoded using features such as scFv identity or affinity, epitope location, hinge and transmembrane domain, costimulatory domain, CD3ζ ITAM number or sequence, promoter strength, CAR expression level, tonic signaling score, tumor antigen density, and manufacturing variables (19, 136, 137, 154, 155, 157, 158). The predicted outputs may include cytotoxicity, cytokine secretion, proliferation, memory phenotype, exhaustion markers, persistence, tumor control, or toxicity-associated readouts. Common model classes include regularized regression, random forests, gradient-boosted trees, support-vector machines, neural networks, and ensemble models (138–142, 158). These approaches are useful because they can detect nonlinear and non-additive interactions among CAR modules, such as cases where the effect of CD28 or 4-1BB costimulation depends on hinge geometry, CAR density, antigen abundance, or ITAM configuration (136, 157, 159).

Structure-aware deep learning models provide another complementary framework, particularly for optimizing the extracellular antigen-recognition domain. Protein language models, graph neural networks, transformer-based models, and structure-prediction tools can help evaluate scFv stability, antigen-binding interfaces, epitope accessibility, aggregation-prone regions, and positively charged surface patches associated with tonic signaling (140, 141, 143). However, predicted binding affinity or developability does not necessarily translate into optimal CAR-T function. Therefore, structure-aware modeling should be integrated with cellular signaling measurements and functional validation assays (138, 143, 158).

### 5.2 High-throughput CAR library screening generates datasets for AI-driven discovery

High-throughput screening technologies have transformed CAR engineering by enabling systematic exploration of large combinatorial design spaces (157, 159, 160). The modular nature of CARs that comprise extracellular binding domains, hinge regions, transmembrane domains, and intracellular signaling motifs, facilitates the construction of diverse receptor libraries through rational recombination of functional modules. As a result, pooled CAR libraries containing hundreds to thousands of variants with distinct signaling architectures are now routinely generated.

Recent studies have employed barcoded CAR constructs to enable parallel screening of intracellular domain combinations (157, 161). In these approaches, each CAR variant is linked to a unique DNA barcode, allowing its identity to be tracked following functional selection. Combinatorial strategies such as signaling motif shuffling and intracellular domain recombination have further expanded the diversity of CAR libraries, enabling systematic interrogation of how different signaling modules influence T-cell behavior (158, 159). Platforms such as pooled screening with single-cell RNA sequencing (scRNA-seq) have provided unprecedented resolution, linking CAR architecture to transcriptional states at the single-cell level (159, 160, 162).

These platforms generate rich datasets that capture multiple dimensions of CAR-T function, including variant enrichment or depletion, proliferation, activation-associated transcriptional programs, cytokine-related gene expression, and differentiation into memory or exhausted states. However, most pooled barcode or single-cell screening approaches do not directly measure cytotoxicity for each CAR variant. Instead, cytotoxic activity is often inferred indirectly from surrogate readouts, such as selective expansion after antigen encounter, depletion of target cells in pooled co-culture, enrichment of cytotoxic effector gene programs, or persistence of specific CAR barcodes after tumor challenge. Therefore, candidate CARs identified in pooled screens require orthogonal validation using arrayed tumor-killing assays, cytokine-release assays, live-cell imaging, flow-based target-cell clearance assays, or *in vivo* tumor-control studies. Single-cell transcriptomic profiling further reveals molecular programs associated with distinct CAR designs, providing insight into pathways governing persistence, activation, and dysfunction (163, 164). Together, these datasets help define quantitative associations between CAR structural features and cellular phenotypes, offering a comprehensive view of CAR-T functional landscapes.

Single-cell and multi-omics datasets are particularly valuable because CARs with similar short-term cytotoxicity may induce distinct long-term cellular trajectories. For example, different CAR architectures may vary in their ability to preserve TCF7-positive stem-like states, avoid TOX/NR4A-associated exhaustion programs, maintain mitochondrial fitness, or resist chronic antigen stimulation (159, 160, 162–164). Dimensionality reduction, clustering, latent-variable modeling, and graph-based methods can organize these high-dimensional profiles into interpretable activation, memory, and exhaustion states (138–140, 159). These models therefore help connect receptor architecture not only to immediate tumor killing, but also to the durability and fitness of the engineered T cell.

However, the complexity of these datasets presents significant analytical challenges. Interactions among signaling domains are often non-additive, with certain combinations producing synergistic or antagonistic effects that cannot be predicted from individual components. Moreover, CAR signaling is context-dependent, influenced by factors such as antigen density, microenvironmental cues, and T-cell intrinsic states. Traditional analytical approaches based on pairwise comparisons or linear models are therefore inadequate to capture the full richness of these high-order interactions.

AI provides a powerful solution to this challenge by enabling the integration and interpretation of high-dimensional datasets (138, 139). Machine learning algorithms can identify patterns across large numbers of CAR variants, uncovering hidden relationships between design features and functional outcomes (142, 158). Dimensionality reduction techniques, such as latent space embedding, can be used to visualize phenotypic landscapes, while clustering methods can classify CAR-T cells into functional states based on transcriptional profiles. Importantly, AI models can learn complex feature interactions, allowing identification of signaling domain combinations that confer desirable properties, such as enhanced persistence or reduced exhaustion.

### 5.3 AI enables predictive and programmable smart CAR-T design

The integration of high-throughput screening data with AI-driven modeling is transforming CAR engineering from a descriptive to a predictive discipline. Machine learning algorithms trained on large-scale CAR datasets can infer relationships between receptor design and functional outcomes, enabling rational selection of CAR architectures with desired properties. This represents a shift from iterative experimental testing toward data-guided design.

One key application of AI is the prediction of T-cell phenotypes based on CAR signaling features. By analyzing datasets derived from combinatorial CAR libraries, machine learning models can identify patterns associated with effective antitumor responses, such as balanced cytokine production, sustained proliferation, and resistance to exhaustion (157, 160). These models can also detect signaling motifs that contribute to tonic activation or dysfunctional states, providing insights into mechanisms underlying CAR-T failure. Importantly, AI can uncover non-intuitive combinations of signaling domains that outperform conventional designs, highlighting the value of data-driven approaches in identifying novel architectures.

Beyond prediction, AI can support iterative CAR optimization. In Bayesian optimization or active-learning workflows, an initial CAR library is screened experimentally, a predictive model is trained on the resulting functional data, and the model then proposes the next set of CAR candidates expected to improve selected objectives (143–145). These objectives may include potent tumor killing, reduced tonic signaling, lower cytokine release, improved persistence, preserved memory phenotype, and selective activation against antigen-high tumor cells (138–140, 158). After each experimental cycle, the model is updated, creating a design-build-test-learn loop that prioritizes the most informative constructs for validation.

AI can also be used to rank and prioritize candidate CAR designs for experimental validation (143). A recent study by Yoshida and colleagues provides a concrete example of AI-guided CAR design by fine-tuning the ESM-2 protein language model with generated CAR sequences and validating predictions against experimentally measured cytotoxicity of CAR-T cells expressing mutated CAR variants (165). This illustrates how protein language models can support CAR sequence–function prediction and candidate prioritization. By integrating multiple performance metrics, including cytotoxicity, persistence, and safety profiles, computational models can identify optimal trade-offs among competing objectives. This is particularly important given that CAR-T efficacy and toxicity are often linked, requiring careful balancing of signaling strength. AI-based optimization approaches, including Bayesian optimization and reinforcement learning, offer the potential to iteratively refine CAR designs by integrating experimental feedback into the design process (144, 145).

AI further enables the development of programmable “smart” CAR-T cells (146, 166, 167). Smart CARs are designed to operate within defined activation thresholds, responding selectively to tumor cells while sparing normal tissues. This can be achieved by tuning parameters such as affinity, receptor density, and signaling strength to create systems that are sensitive to high antigen density but remain inactive in low-density contexts. AI models can predict these threshold behaviors, enabling design of CARs with improved specificity and reduced off-tumor toxicity.

Interpretability is also critical for clinical translation. Black-box predictions alone are insufficient because CAR-T products require mechanistic justification, reproducible manufacturing, and regulatory evaluation. Feature-importance analysis, SHAP values, sensitivity analysis, partial-dependence analysis, and counterfactual design can help identify which receptor features drive model predictions. For example, these analyses may reveal that a given costimulatory domain improves persistence only when paired with a specific hinge or lower CAR surface density, or that tonic signaling risk is driven more by scFv charge distribution than by costimulatory domain choice.

Looking forward, the combination of AI, high-throughput screening, and synthetic biology is poised to accelerate the next generation of CAR-T innovation. Integration of multi-omics data including transcriptomic, epigenomic, and proteomic profiles, will further enhance predictive accuracy, while advances in protein engineering and structural modeling will refine antigen recognition and specificity. Ultimately, AI is positioned to serve as a central design engine for CAR-T engineering, enabling systematic exploration of complex design spaces and accelerating the development of safer, more effective, and increasingly personalized cell therapies.

## 6 Conclusion and future perspectives

CAR-T cell therapy has evolved from a conceptual extension of T cell receptor signaling into a highly customizable synthetic platform. The central insight emerging from this body of work is that CAR function is fundamentally governed by signaling dynamics rather than receptor structure alone. As highlighted throughout this review, CAR signaling requirements are inherently temporal and context-dependent: strong and amplified signaling is required during the initial phase of tumor engagement, whereas sustained or excessive signaling becomes detrimental, driving exhaustion, metabolic dysfunction, and treatment-related toxicities.

This duality gives rise to a unifying design principle: effective CAR-T therapy requires the integration of “more is better” and “less is more” strategies within a single system. Signal amplification through proximal signaling engineering, cytokine armoring, and enhanced trafficking enables CAR-T cells to overcome early barriers such as low antigen density, poor tumor infiltration, and immunosuppressive microenvironments. In contrast, signal restraint achieved through structural tuning, transcriptional and epigenetic reprogramming, metabolic optimization, and dynamic control circuits preserves long-term persistence and prevents premature exhaustion. These approaches are not opposing, but rather sequential and complementary, reflecting distinct phases of the CAR-T response.

Importantly, the modular nature of CARs provides an unprecedented opportunity to engineer signaling at multiple levels simultaneously. Rather than relying on single modifications, next-generation CAR-T cells are likely to incorporate multi-layered designs, combining receptor-level tuning, cytokine and microenvironmental modulation, and programmable control systems. Such integrated approaches will enable CAR-T cells to adapt dynamically to tumor evolution and maintain functional fitness over time.

The emergence of high-throughput screening and artificial intelligence further accelerates this transition from empirical design to predictive engineering. By mapping the complex relationships between CAR architecture, signaling strength, and functional outcomes, AI-driven models can identify optimal signaling configurations that balance efficacy, persistence, and safety. These advances raise the possibility of precision CAR-T therapy, in which receptor design is tailored to tumor antigen density, microenvironmental context, and patient-specific characteristics.

Despite these advances, several challenges remain. Achieving precise spatial and temporal control of signaling, minimizing toxicity while maintaining potency, and translating complex engineered systems into clinically scalable products will require continued innovation. In addition, tumor heterogeneity, antigen escape, and immunosuppressive niches remain formidable barriers, underscoring the need for combinatorial and adaptive strategies.

In summary, CAR-T therapy is transitioning from a static receptor-based approach to a programmable signaling system. Future progress will depend on the ability to define and implement optimal signaling trajectories rather than simply maximizing activation. By integrating advances in synthetic biology, immunology, and computational modeling, next-generation CAR-T cells have the potential to achieve durable, safe, and broadly applicable antitumor responses across diverse malignancies.
